# Inflammatory Episode in a Patient With Probable Iatrogenic Cerebral Amyloid Angiopathy: An ARIA Before the Storm?

**DOI:** 10.1111/ene.70298

**Published:** 2025-10-21

**Authors:** Ulf Jensen‐Kondering, Gregor Kuhlenbäumer, Nils G. Margraf

**Affiliations:** ^1^ Department of Neuroradiology University of Schleswig‐Holstein, Campus Lübeck Lübeck Germany; ^2^ Department of Radiology and Neuroradiology University of Schleswig‐Holstein, Campus Kiel Kiel Germany; ^3^ Department of Neurology University of Schleswig‐Holstein, Campus Kiel Kiel Germany

**Keywords:** amyloid, ARIA‐E, iatrogenic CAA, inflammatory CAA, MRI

## Abstract

**Introduction:**

Amyloid related imaging abnormalities effusion/edema (ARIA‐E) is seen in patients treated with antiamyloid antibodies. It resembles cerebral amyloid angiopathy (CAA) related inflammation (CAA‐ri) caused by an inflammatory response to amyloid deposition in the walls of cortical and leptomeningeal vessels in patients with sporadic CAA. Recently, temporary inflammatory imaging findings that remained clinically silent have been described in patients with iatrogenic CAA (iCAA).

**Results:**

We describe a case of probable iatrogenic CAA (iCAA), which demonstrated radiological features of CAA‐ri that remained clinically silent and spontaneously resolved before the inaugural intracranial hemorrhage. Clinical and radiological features resembled ARIA‐E.

**Discussion:**

This case adds to the clinical and radiological spectrum of iCAA and suggests an immune‐mediated response to amyloid deposition.

## 1 Introduction

Various imaging patterns have been described in iatrogenic cerebral amyloid angiopathy (iCAA). Typical imaging markers overlap with those of sporadic CAA, namely intracranial hemorrhages, cerebral microbleeds (CMB), convexity subarachnoid hemorrhage, cortical superficial siderosis (cSS), white matter hyperintensities, and enlarged perivascular spaces [1]. Recently, imaging features suggesting inflammation have been described [2].

## 2 Methods

We present a male patient with probable iCAA previously described by us [3]. In short, he had neurosurgery at the age of 3 months due to an intracranial aneurysm, including implantation of a lyophilized dura graft. He was clinically and radiologically followed up for ongoing seizures. There was an increasing load of CMB, white matter lesions, cSS, enlarged perivascular spaces, and he had a first ICH at the age of 35 years.

**FIGURE 1:**
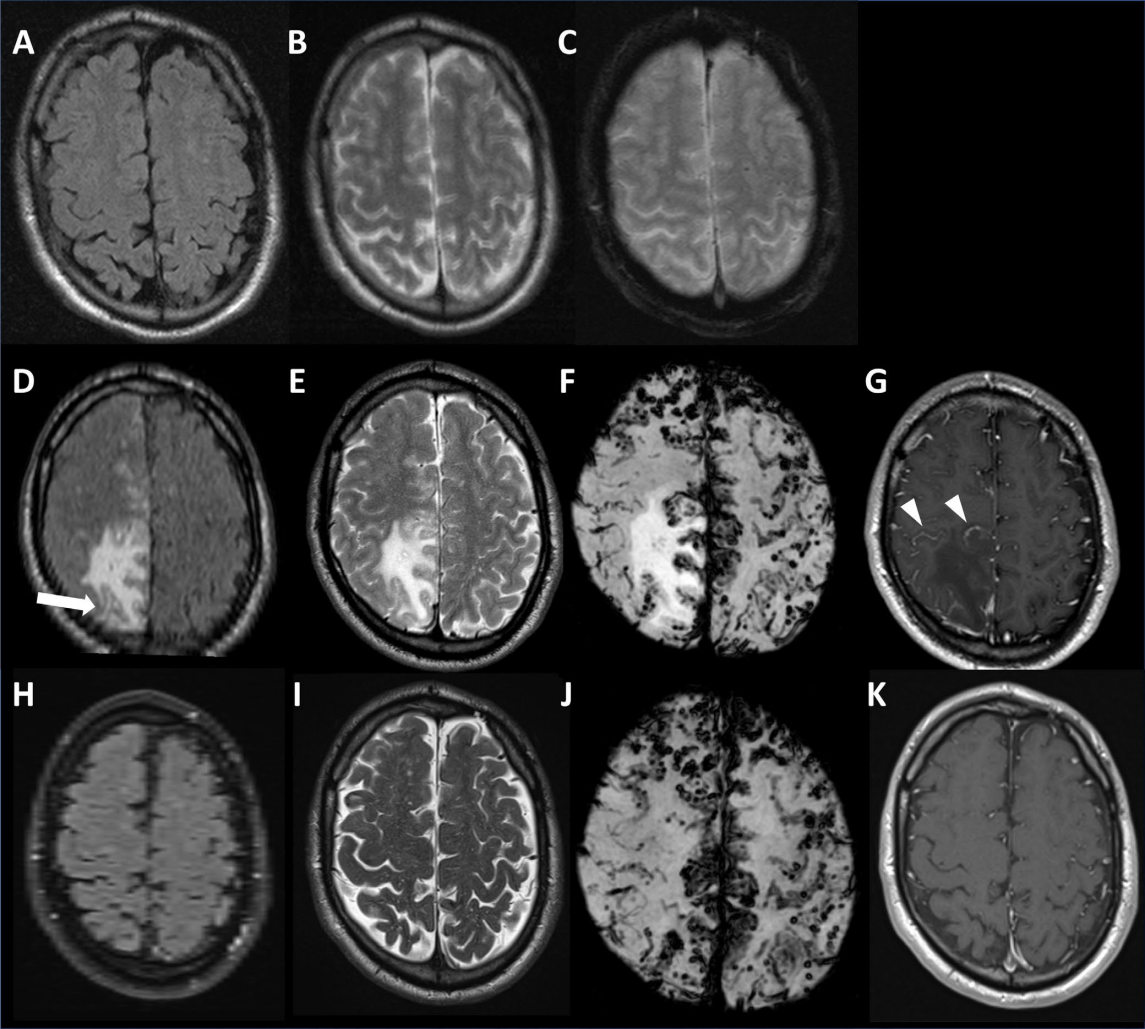
Evolution of the imaging features 7 years before (A–C), during (D–G) and after (H–K) the inflammatory episode. During the episode, the right parietal lobe demonstrates focal swelling, vasogenic edema on FLAIR (D) and T2w (E), adjacent sulcal hyperintensity (arrowhead) on FLAIR (D) and leptomeningeal enhancement (arrowhead) on contrast enhanced T1w (G). Note complete resolution (H–K) after 6 months follow‐up. T2* (C) and SWI (F, J) depicting the hemorrhagic markers on corresponding slices.

Eighteen months before the inaugural ICH, he had an episode of temporary occurrence of imaging features compatible with inflammation during routine MRI follow‐up. While a previous MRI did not demonstrate these findings (Figure 1A–C), we now detected the presence of vasogenic edema (in the absence of restricted diffusion), proteinaceous effusion into the subarachnoid space (characterized by hyperintense effacement of the sulci on FLAIR) and leptomeningeal contrast enhancement compatible with break‐down of the integrity of the brain blood barrier and leakage into the surrounding tissue. An inflammatory process was favored over infarction, venous congestion, peritumoral edema, and true inflammation since no cytotoxic edema was present and no vessel occlusion or tumor could be demonstrated (Figure 1D–G). No change in the clinical condition was observed. The patient had an APOE ε 3/4 genotype, that is, possessed one APOE ε4 allele. No biomarkers in peripheral blood or CSF were obtained at the time, and he was followed clinically, and no treatment was initiated. On follow‐up MRI, the imaging abnormalities completely resolved, and no obvious hemorrhagic sequela could be detected (Figure 1H–K).

## 3 Discussion

Iatrogenic CAA is caused by deposition of amyloid in leptomeningeal vessels, inoculated by cadaveric dura mater materials or neurosurgery itself and hypothesized to propagate through the brain in a prion‐like manner [1].

Inflammatory imaging features have been described before in patients with iCAA. Fandler‐Höfler et al. Reported imaging features suggesting inflammation in 27.4% in a cohort of 51 iCAA patients [2]. Most of them were asymptomatic and temporary. A case of CAA‐ri in a patient with iCAA has been described by Banerjee and coworkers. However, the patient was symptomatic and presented after the inaugural ICH had taken place [4].

The imaging and clinical features described in our patient closely resemble ARIA‐E (amyloid‐related imaging abnormalities‐edema/effusion) seen in patients with antiamyloid treatment [5]. The exact mechanism of this phenomenon in iCAA is unknown, but it may be reasonable to assume a local reaction of the brain parenchyma and vessels in the process of excretion of superfluous amyloid. In CAA‐ri, autoantibodies are thought to induce a perivascular inflammation facilitating amyloid removal [6, 7, 8]. ARIA‐E is characterized by an immune‐mediated vascular and perivascular response to amyloid breakdown products during treatment with antiamyloid antibodies. ARIA‐E closely resembles CAA‐ri in terms of neuroradiological presentation, with the presence of vasogenic edema, proteinaceous effusion, and leptomeningeal contrast enhancement [9]. While most ARIA remain clinically silent, CAA‐ri requires a defined clinical presentation such as headache, decreased consciousness, behavioral change, focal neurological signs, or seizures [10]. Both conditions are associated with the presence of the APOE ε4 allele [11].

## 4 Conclusion

This case adds to the expanding spectrum of clinical presentation and imaging findings in iatrogenic CAA. More studies exploring the potentially autoimmune character of this phenomenon should be conducted.

## Author Contributions

Ulf Jensen‐Kondering, Gregor Kuhlenbäumer, and Nils Margraf contributed to the study conception and design. Material preparation, data collection, and analysis were performed by Ulf Jensen‐Kondering, Gregor Kuhlenbäumer, and Nils Margraf. The first draft of the manuscript was written by Ulf Jensen‐Kondering, and all authors commented on previous versions of the manuscript. All authors read and approved the final manuscript.

## Ethics Statement

The authors have nothing to report.

## Consent

Consent to Participate: The legal guardian of the patient gave consent for retrospective analysis of the data. Consent for Publication: The legal guardian of the patient gave consent for publication.

## Conflicts of Interest

The authors declare no conflicts of interest.

## Data Availability

All relevant data can be found within the manuscript. Specific data is available upon reasonable request.
